# Medical Department, University of Pennsylvania, Raises Its Requirements for Admission

**Published:** 1907-03

**Authors:** Charles H. Frazier

**Affiliations:** Philadelphia


### Medical Department, University of Pennsylvania, Raises its Requirements for Admission.

*Editor Buffalo Medical Journal*:

Sir:—I take the liberty of sending you a statement of the requirements for admission to the Medical Department of the University of Pennsylvania, which have recently been adopted by the Board of Trustees.

According to the plan finally adopted, the requirements will be increased gradually beginning with the annual session in September, 1908, and reach the maximum September, 1910. The present requirements cover four years graded course in a High School or its equivalent. The essential points in the new requirements are as follows:

I. For the session 1908-1909, in addition to the present requirements, either one of two foreign languages, French or German; (2) Physics; (3) Inorganic Chemistry, including qualitative analysis; (4) General Biology or General Zoology.

II. For the session of 1909-1910, in addition to the requirements of 1907-1908, the candidate must have completed successfully work equivalent to that prescribed for the Freshman Class in Colleges recognised by this University.

III. For the session 1910-1911, in addition to the requirements of 1907-1908, candidates must have completed successfully work equivalent to that prescribed for the Freshman and Sophomore Classes.

IV. Candidates who have successfully completed at least three years of an accepted College Course, may be admitted with conditions in Chemistry, Physics and General Biology or Zoology.

The maximum requirement is two years of collegiate training, including Biology, Chemistry and Physics. The two additional years of Collegiate training are regarded as an adequate preparation for entrance into a professional school. The importance of the candidate having had instruction in subjects leading to the study of Medicine is so generally recognised that Biology, Chemistry and Physics have been added to the requirements. This combination of special and general training is without doubt a much more logical requirement than a Collegiate degree which does not imply any special preparation for medical studies.

Charles H. Frazier.

Philadelphia, February 15, 1907.

---

The Danger of Dust as a Cause of Tuberculosis.— Dr. George Homan, of St. Louis, contributed a paper on this subject at the recent annual meeting of the Mississippi Valley Medical Association in which he submitted the following points: (1) That efforts toward the eradication of human tuberculosis will fail which do not take full account of household dust as a factor in the dissemination of that disease; (2) that scientific tests have shown that the seeds of pulmonary tuberculosis, harbored within doors in the dried state, are capable of retaining their effective vitality for prolonged periods of time; (3) that any method or procedure employed in inhabited buildings which causes dust to be disseminated must be considered as tending to spread the seeds of consumption; (4) that hotels, clubs, theaters, office buildings, schools, churches, and business establishments generally should be required by law to introduce and operate dustless methods of cleaning. This part of their mechanical equipment should be held as necessary as provisions similarly made for warming, ventilation, and for fire protection and fire escape. The employment of dustless methods in private residences is urged as being equally imperative for the control and suppression of all forms of tuberculous disease.—*Medical Fortnightly.*

---

Reporter—To what do you attribute your great age?

Oldest Inhabitant—I bain't sure yet, sir. There be several o' them patent med'cine companies as is bargainin' with me.

